# Live Cells Imaging and Comparative Phosphoproteomics Uncover Proteins from the Mechanobiome in *Entamoeba histolytica*

**DOI:** 10.3390/ijms24108726

**Published:** 2023-05-13

**Authors:** Gagan Deep Jhingan, Maria Manich, Jean-Christophe Olivo-Marin, Nancy Guillen

**Affiliations:** 1 Institut Pasteur, Cell Biology of Parasitism Unit, 75015 Paris, France; gaganjhingan@hotmail.com (G.D.J.); maria.manich@pasteur.fr (M.M.); 2 Institut Pasteur, Biological Image Analysis Unit, 75015 Paris, France; jean-christophe.olivo-marin@pasteur.fr; 3 Centre National de la Recherche Scientifique, CNRS UMR3691, 75015 Paris, France; 4 Centre National de la Recherche Scientifique, CNRS-ERL9195, 75015 Paris, France

**Keywords:** *Entamoeba histolytica*, motility, phosphoproteomics, mechanobiome

## Abstract

*Entamoeba histolytica* is a protozoan parasite and the causative agent of amoebiasis in humans. This amoeba invades human tissues by taking advantage of its actin-rich cytoskeleton to move, enter the tissue matrix, kill and phagocyte the human cells. During tissue invasion, *E. histolytica* moves from the intestinal lumen across the mucus layer and enters the epithelial parenchyma. Faced with the chemical and physical constraints of these diverse environments, *E. histolytica* has developed sophisticated systems to integrate internal and external signals and to coordinate cell shape changes and motility. Cell signalling circuits are driven by interactions between the parasite and extracellular matrix, combined with rapid responses from the mechanobiome in which protein phosphorylation plays an important role. To understand the role of phosphorylation events and related signalling mechanisms, we targeted phosphatidylinositol 3-kinases followed by live cell imaging and phosphoproteomics. The results highlight 1150 proteins, out of the 7966 proteins within the amoebic proteome, as members of the phosphoproteome, including signalling and structural molecules involved in cytoskeletal activities. Inhibition of phosphatidylinositol 3-kinases alters phosphorylation in important members of these categories; a finding that correlates with changes in amoeba motility and morphology, as well as a decrease in actin-rich adhesive structures.

## 1. Introduction

Moving cells sense mechanical stimuli from their environment and recruit diverse cytoskeletal components into specific subcellular compartments necessary to build the intracellular contractility network, which allows them to respond to stimuli [1,2]. The network of macromolecules involved in cell responses to external signals and regulation of cell motility is known as the mechanobiome. In eukaryotic cells, the receptors mediating adhesion of cells to the extracellular matrix (ECM) are integrins, which recognize biochemical, mechanical, and topographical cues from the extracellular milieu [3]. Integrins consist of two non-covalently linked α and β subunits which bind to specific motifs (e.g., the RGD tripeptide) located on fibronectin, vitronectin, and laminin. ECM-activated integrins connect the actin microfilaments of the cytoskeleton to ECM components via adaptor proteins, which build cell adhesion structures such as focal adhesions (FAs) and invadosomes [3]. 

Hundreds of proteins which modulate cell responses depending on the nature of a stimulus constitute the FAs (roughly 10 µm size at mature stage). These include scaffolding and structural proteins, kinases, and phosphatases [4]. Central to the protein composition and dynamics of FAs are talin, α-actinin, and kindlin, which all bind to the β-integrin cytoplasmic tail. Specific domains of talin interact with actin, vinculin, and paxillin, acting as a docking platform for the many other FAs components [5] needed for signalling cell responses. Signalling molecules within FAs include the focal adhesion kinase (FAK), small GTPases, as well as Rho-associated coiled-coil-containing protein kinase (ROCK).

Invadosomes are also able to degrade the ECM through local enrichment and secretion of matrix-lytic enzymes such as matrix metalloproteinases [6], allowing the cells to penetrate the meshwork of the ECM or to cross tissue boundaries. Depending on the cell type, invadosomes can derive from podosomes (macrophages, dendritic cells, or osteoclasts) or invadopodia (cancer cells). Podosomes are found in highly motile cells (~1 μm in size), and are organized into three substructures under the membrane cortex: core, ring, and cap [7]. They are able to sense and report specific ECM properties such as stiffness and topography [8]. Podosomes, initially described as dot-like enrichments in F-actin, consist of more than 300 different components. They have a protein complexity comparable to that of FAs [9]; indeed, the two proteomes share common elements [10]. 

The core of podosomes consists of branched microfilaments generated by the ARP2/3 complex and actin regulators such as (N)-WASP, WIP, CDC42, cortactin, and cofilin. A ring of adhesion-related proteins (e.g., vinculin, talin, paxillin, and PYK2) surround the core [6]. The cap consists of cross-linked and bundled actin microfilaments that encapsulate the core; this network includes actin-binding proteins such as cross-linkers (α-actinins and LSP1), bundlers (supervillin and fascin), nucleators (formins), and depolymerization factors (gelsolin) [8]. Actomyosin filaments compose cables that connect individual podosomes into superstructures such as clusters in macrophages, rosettes in endothelial cells or fibroblasts, and rings or peripheral belts in osteoclasts [6]. Phosphatidylinositol 3-kinases (PI3Ks), a family of lipid kinases, interacts with FAs or podosome proteins [11] and is required for cell survival, metabolism, and motility through the production of lipid second messengers [12]. 

In this work, we have determined a critical role of protein phosphorylation status and its effects on mechanobiome components in the human pathogenic amoeba *Entamoeba histolytica*. This amoeba migrates from the intestinal lumen to different compartments of the human body depending on the stage of infection, leading to amoebiasis: a common intestinal infectious disease [13]. Podosome-like structures are common in *E. histolytica*, whether the amoebae are resting, moving, seeded on glass [14], or located on ECM components [15,16,17]. F-actin is enriched within these subcellular compartments in the form of fibers, dots, and rings, all of which contain ARP2/3 components [14]. 

As in other motile cells, polarization in *E. histolytica* is a complex phenomenon in which changes occur on the membrane surface through an initial exploratory step characterized by pseudorandom blebbing [18]. This is followed by pseudopodium growth that guides displacements at the cell front [19]. The molecular and environmental mechanisms that support the switch from blebbing to pseudopod extension in *E. histolytica* are not known. However, protrusion of a pseudopod is intimately coupled to membrane folding at the cell rear in a superstructure known as an uropod, which concentrates the actomyosin cytoskeleton and actin-binding proteins [20]. The activity of several proteins has been described in amoebae polarization dynamics, including myosin II [21,22], filamin 2/ABP120 [23,24], and α-actinin [25]. Signal transduction factors activated during amoebic polarization include the small GTPases RacG [26], RacA [27], Rab A [28], and some of their corresponding GTP exchange factors [29,30,31]. 

The signalling pathways activated during amoebic displacements involve kinases, including PAK [32,33] and PI3Ks [25,34]. The fact that lipid rafts and the signalling lipid PI (4,5)P2 localize to the uropod [35] makes PI3Ks a central factor regulating motility in *E. histolytica.* Therefore, we hypothesized that modifications in PI3K activities would help to identify components of the amoebic mechanobiome. We used wortmannin (Wtmn), a drug that penetrates living cells and inhibits PI3Ks. The cell morphology of trophozoites and actin-rich structures, as well as the kinetics of cell motility, were modified under inhibition of PI3Ks. Phosphoproteomic studies determined a set of more than a thousand globally and quantitatively phosphorylated proteins. Systematic cataloguing of the resulting data and bioinformatics analyses allowed us to identify significant enrichment of signalling and structural molecules involved in vesicle trafficking, cytoskeleton, and DNA modification pathways. Wortmannin treatment modified the phosphorylation dynamics of important members of the phosphoproteome, including signalling and structural molecules involved in cytoskeleton dynamics, leading to changes in adhesive structures in *E. histolytica*. 

## 2. Results

### 2.1. Kinetic Parameters of E. histolytica Motility in the Presence of Wortmannin

**Figure 1:**
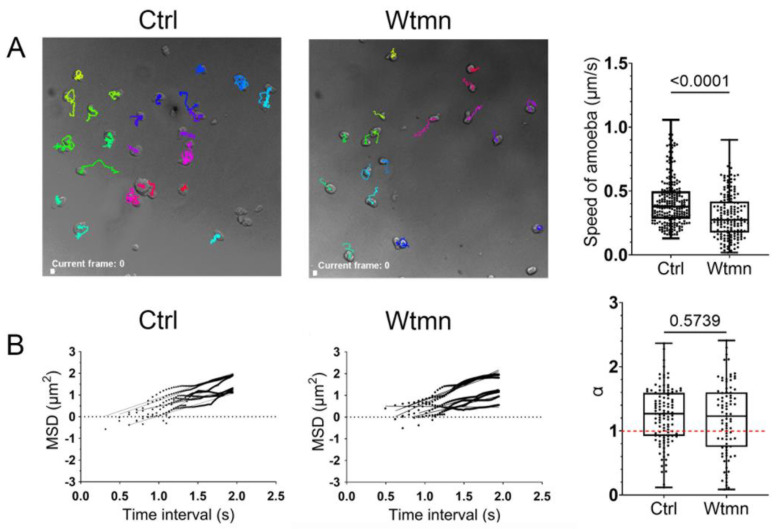
Kinetic parameters of *E. histolytica* motility after treatment with wortmannin. (**A**) Micrographs of live amoebae migrating on a glass surface that were treated or untreated with Wtmn. The video microscopy was acquired with a spinning disk microscope. Sequences of images (65 frames) were visualized and analysed with Icy software, allowing registration of the cells’ tracks. The current image is an example of the trajectories. The white square scale bar indicates 10 µm. The speed of the amoeba was determined from the tracks of individual cells in track manager and motion profiler plugins in Icy. The entire sequence of the video microscopy is displayed in Supplemental Videos S1 and S2. The speed of cells was decreased by the Wtmn treatment (*p* = 0.0001). (**B**) Examples of mean square displacements for Wtmn-treated and untreated trophozoites. The video microscopies were obtained from moving trophozoites. The MSD analysis was conducted on video sequences of 65 s for each condition. The trajectories and MSDs of independent amoebae (*n* = 227 cells for the control and *n* = 166 cells for Wtmn-treated) were obtained as described in material and methods section. The MSD and the corresponding time data were plotted as Log (MSD(T)) = f(Log(T(s)), and the slope for each individual plot (85 plots for control cells and 111 plots for Wtmn-treated cells) was obtained using a regression graph in Prism. The mean of data (α > 1) indicates superdiffusive displacements for the two populations of trophozoites. Ctrl = control cells, Wtmn = treated cells.

Wortmannin (Wtmn) is a fungal metabolite that inhibits phosphatidylinositol 3-kinases (PI3Ks) activity [36], which regulates chemotaxis [25] and mechanotransduction pathways [34] in *E. histolytica*. The powerful performance of live cell imaging allows us to characterize the global effects of Wtmn on amoeba motility. We examined populations of trophozoites seeded on glass and in the presence or absence of a sublethal dose of Wtmn. The trajectories of the trophozoites were determined using time-lapse video microscopy with Icy software plugins [37], as described in the methods section. Trophozoites from the control condition could migrate relatively long distances, with a median speed of 0.38 µM·s^−1^ (*n* = 227 cells). In comparison, trophozoites incubated in the presence of Wtmn moved with a reduced median speed of 0.28 µm·s^−1^ (*n* = 166 cells, *p* = 0.0001) (Figure 1A and Supplemental Movies S1 and S2).

The trajectories of the trophozoites allowed us to calculate the mean-square displacement (MSD), which, in the context of cell migration, is a measure of the surface area explored by cells over time. MSD relates to the overall efficiency of migration [38] and determines whether cell displacements are stochastic. The α-values correspond to the slopes of curves derived from log plots of MSD over time (Figure 1B). It is thus an index of random or directional persistence of motility. Wtmn treatment did not change the motion mode of trophozoites since their displacements were not Brownian, confirming our previous findings [39]. The median values of α corresponded to α = 1.3 (*n* = 111 α) for the control and α = 1.2 (*n* = 86 α) for Wtmn-treated amoebae (Figure 1B), indicating superdiffusive motion in both conditions whose displacement mode were not statistically different.

### 2.2. Cell Surface Morphology and Adhesive Structures in E. histolytica following Wortmannin Treatment

**Figure 2:**
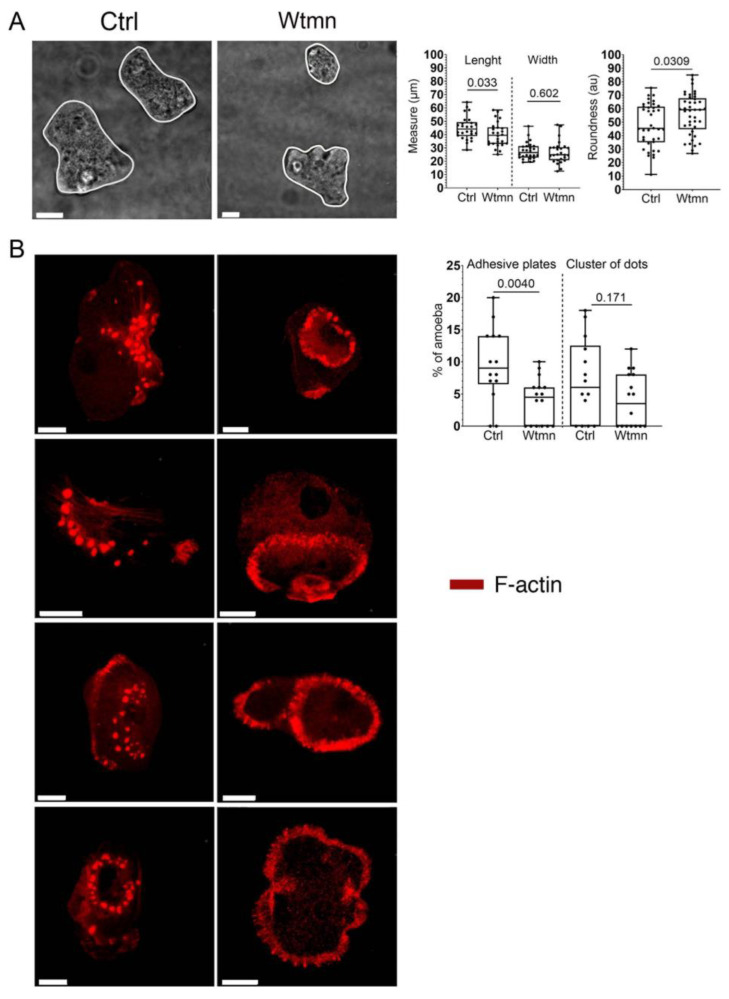
Cell surface morphology and intracellular adhesive structures in *E. histolytica*. (**A**) Bright field micrographs of fixed amoebae treated with or without Wtmn were acquired using a confocal microscope. The entire cells were segmented and considered as an independent ROI (ellipse). Using the active contours plugin, the automatic quantification of length (first axis), width (second axis), and roundness (total contour) of trophozoites was determined. Scale bar is 10 µm. The length of cells decreased in the presence of Wtmn, which correlated with an increase in their roundness (graph). (**B**) Localisation of F-actin in fixed trophozoites. The micrographs show representative adhesive structures containing F-actin decorated by TRITC-phalloidin (red). In the left column, clusters and rings of dots are visualized. In the right column, adhesive plates of different sizes beneath the cell surface are visible. Scale bar = 10 µm. Images derived from 14 and 16 confocal planes in 302 counted control cells and 283 counted cells treated with Wtmn, respectively, allowed us to summarize the percentage of amoebae with actin-rich structures. For adhesive plates (*n* = 25 cells), 9% out of 302 cells were positive in control conditions, and 4.5% (*n* = 21 cells) out of 283 cells were positive in Wtmn-treated conditions. Overall, 3.5% of Wtmn-treated parasites (*n* = 10 cells out of 283) presented with clusters of dots, compared to 6% of control parasites (*n* = 16 cells out of 302). The graph shows a reduction in adhesive plates (50%) and clusters of dots (40%) in Wtmn-treated trophozoites presenting structures.

To determine morphology changes following the incubation of trophozoites with a low dose of Wtmn, we scored the length, width, and roundness of the parasites (Figure 2A,B). The cell borders were determined by the active contour analysis plugin in Icy, and the entire cells were considered as the region of interest (ROI = ellipse). The median measure of the first axis (length) decreased by 10.5% (*p* = 0.03) for Wtmn-treated parasites (*n* = 26 cells, 39.5 µm) compared to the control (*n* = 26 cells, 44.1 µm). The median measure of the second ellipse axis (width) was reduced by a factor 6%, which was not significant (*p* = 0.6) for Wtmn-treated parasites (*n* = 26 cells, 24.9 µm) compared to the control (*n* = 26 cells, 26.5 µm). The median ellipse roundness increased by a factor 1.3 (*p* = 0.03) for Wtmn-treated parasites (*n* = 41 cells, 59.2 au) compared to the control (*n* = 38 cells, 45.3 au). The data indicates slight but significant morphological changes induced by the presence of Wtmn, with an increase in the roundness of trophozoites correlating with a decrease in length. 

Adhesive plates and dots structures were counted in fixed trophozoites using indirect fluorescence of F-actin decorated by TRITC-phalloidin. The percentage of amoebae with at least one adhesive plate was reduced by 50% (*p* = 0.004) for Wtmn-treated parasites compared to the control. The percentage of amoeba with a cluster of dots was reduced to 41.2% (*p* = 0.17) for Wtmn-treated parasites. Treatment of *E. histolytica* with Wtmn decreased the number of adhesive structures (clusters of dots and adhesive plates) within the trophozoites. 

Overall, these data reinforce the notion that the inhibition of PI3 kinases by Wtmn has an effect on the kinetic parameters, morphology, and adhesive structures of *E. histolytica*, in line with previous finding concerning amoebic PI3Ks [25,34,35], and confirmed here using a sublethal dose of Wtmn.

### 2.3. Deciphering the Phosphoproteome of Growing E. histolytica 

**Figure 3:**
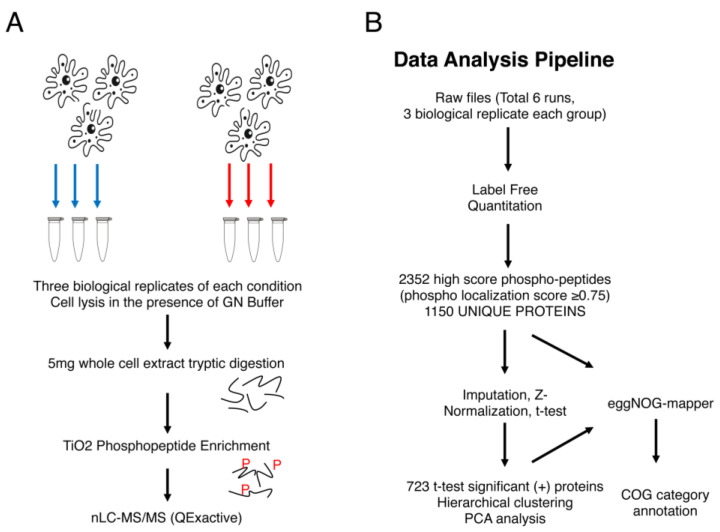
Phosphoproteome analysis workflow of Wtmn-unstimulated and stimulated *E. histolytica* trophozoites. (**A**) Schematic illustration of the design and sample processing workflow used to perform the quantitative phosphoproteomics analysis across the two conditions. Cell pellets were lysed with 6 M GN Buffer, and 5 mg of lysates were trypsin-digested. The purified peptides were further used for a TiO_2_-based phosphoenrichment process. Relative quantitative phosphoproteomics was performed via high-resolution LC-MS/MS analysis using a QExactive instrument. (**B**) Workflow for proteomics data analysis. The six mass spectrometry raw files were analysed using MaxQuant against the *E. histolytica* UniProt reference proteome. LFQ abundance and statistical tests (see methods) determined 1150 unique proteins carrying at least one phosphorylated amino acid. Among these, the abundance of 723 proteins was modified by Wtmn treatment. Functional annotations in categories were performed using the eegNOG mapper.

To identify proteins related to the activity of the PI3Ks in absence of cell death, trophozoites were treated with Wtmn, and non-treated cells were used as controls for global phosphoproteomics enrichment analysis. The amoebic protein phosphorylation profile was determined in cell extracts obtained in three independent biological experiments in each growth condition (methods section), utilizing a TiO_2_-based phosphoenrichment mass spectrometry approach for a total of six phosphoenrichment experiments (Figure 3). Statistical and bioinformatic approaches were further applied for quality control of the data, as well as to identify and annotate all the identified proteins into various functional classes.

**Figure 4:**
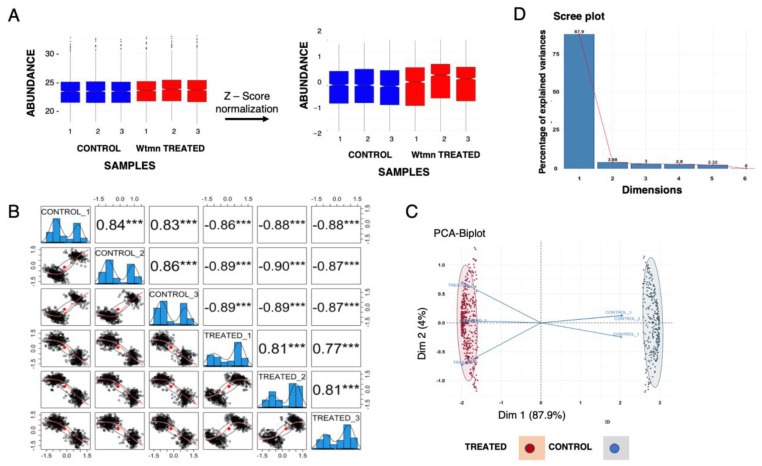
Data summary and multivariate statistical analysis of the identified phosphopeptides. (**A**) Boxplot representing the distribution of peptide intensity abundance values before and after Z-score normalization. The blue and red box plots represent the control, and Wtmn-treatment peptide intensities, respectively, and the notches represent 95% confidence intervals for each median. (**B**) Pairwise scatter plots of normalized *t*-test significant phosphopeptide intensity across control and Wtmn-treatment biological replicates and their corresponding Pearson correlation scores. The *** indicates statistically significant channels. (**C**) Biplots of a principal component analysis performed on *t*-test significant phosphopeptide intensity across control and treatment biological replicates. All 723 significant proteins were used in the analysis. (**D**) The scree plot for the principal components displaying the eigenvalues in a downward curve, ordering the eigenvalues from largest to smallest.

The raw data representing identification and relative quantitation of the identified phosphopeptides were further analyzed (Figure 4A) for quality and statistical control following LC-MS/MS stringency methods including log2 transformation of LFQ intensities, filtering, normalization, and *t*-tests across the two sample groups (Figure 4B). Principal component analysis of the data (Figure 4C) showed the reproducibility of the experimental approach among the biological replicates and explained the observed data variance among the two growth conditions. In total, 2352 phosphorylated peptides were identified in the composite phosphoproteome at the 1% FDR stringency level (Supplemental Table S1, sheets 1 to 5), which matched 1150 unique phosphoproteins in the AmoebaDB database (Supplemental Table S1, sheet 2). These proteins are referred to as the phosphoproteome from *E. histolytica* in this study. 

Fractions of variability in the data captured by each of the principal components are represented by bars (Figure 4D). The cumulative variance captured by the principal components is shown as a line. The first two principal components together account for more than 90% of the variance.

**Figure 5:**
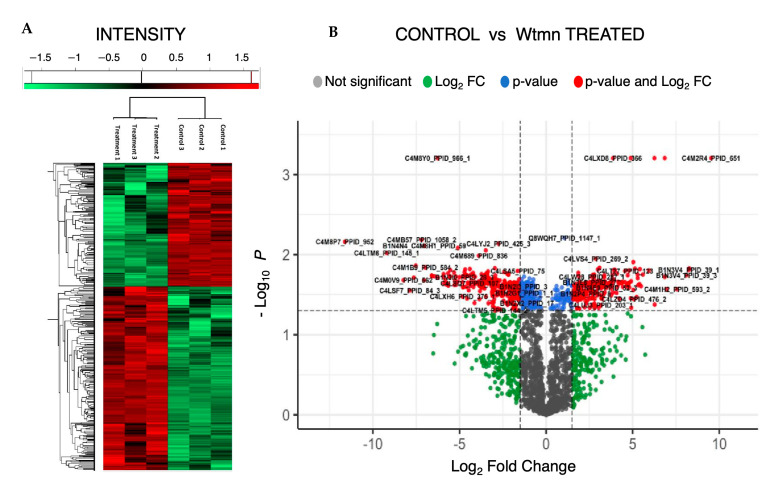
Clustergram and univariate analysis of statistically significant proteins. (**A**) Expression map of consistently measured 723 *t*-test significant phosphopeptides among the two conditions. The Z-scored normalized abundance values of proteins are represented by red (high abundance) and green (low abundance) colors, as indicated in the color scale bar at the top. Hierarchical clustering was based on Euclidean distance, average linkage of normalized expression, and pre-processing with k-means. (**B**) Volcano plot of control vs. treatment. The dashed vertical lines highlight linear fold-changes greater than 1.5, and the solid horizontal line highlights the Storey-adjusted *p*-value cut-off of 0.05. Significant and non-significant proteins are represented by different colors.

The obtained data set corresponds to 14% of the estimated *E. histolytica* proteome, consisting of 7958 unique proteins (UniProtKB data base, reference UP000001926). Further statistical testing of the data (Supplemental Table S1, sheets 3 and 4) showed significant abundance changes for 723 phosphopeptides from the total identified total phosphoproteome for the two growth conditions. (Supplemental Table S1, sheet 2). Clustering using a heat map (Supplemental Table S1, sheet 5) and volcano plot reveals differential and significant abundance changes (FC > 1.5, *p*-value cut-off of 0.05) in the phosphoproteome during Wtmn treatment (Figure 5A,B).

### 2.4. Functional Annotation of the E. histolytica Phosphoproteome

**Figure 6:**
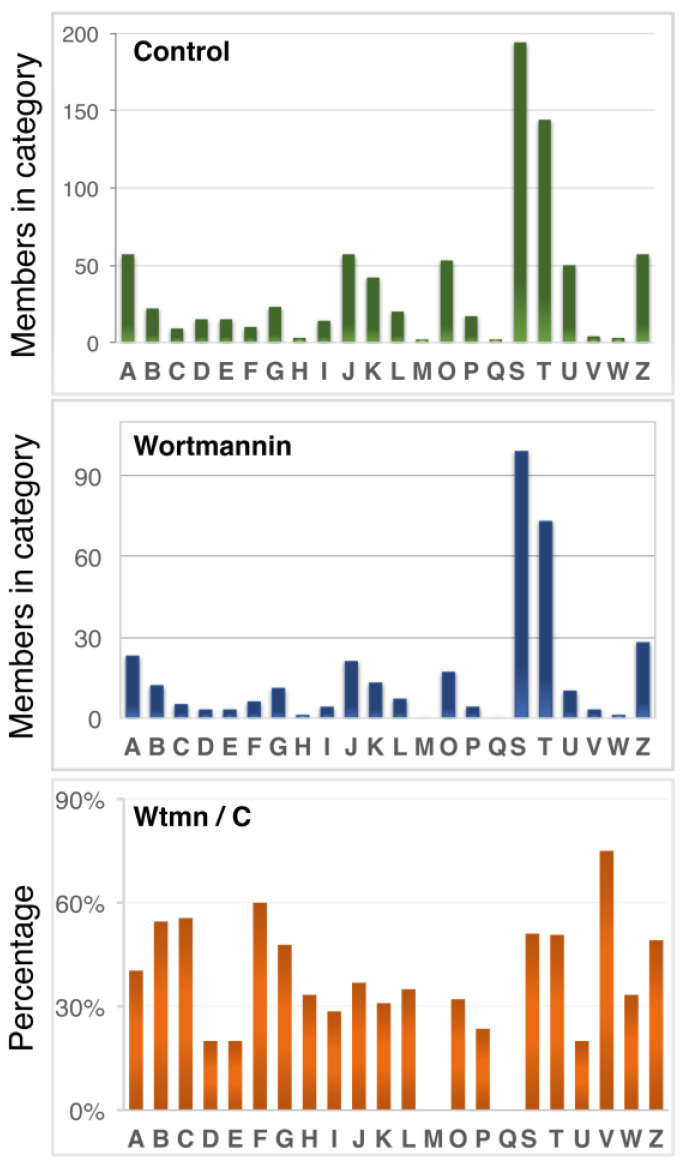
Functional categories of proteins present in the phosphoproteome of *E. histolytica*. Predicted proteins were assigned to clusters of orthologous groups of proteins (COG) in the eggNOG database using the eggNOG-mapper V.2 (http://eggnog-mapper.embl.de/, accessed on 1 April 2022) according to their defined parameters (hit e-values ≤ 1 × 10^−3^). (**Upper panel**) Categories of orthologous groups found in the global prosphoproteome dataset control. (**Central panel**) Categories of orthologous groups found with changes in phosphorylation in the Wtmn-treated dataset. (**Lower panel**) Percentage of proteins modulated by Wtmn treatment in relation to the control. The one-letter abbreviations for the functional categories correspond to COG–NCBI (https://www.ncbi.nlm.nih.gov/research/cog, accessed on 1 April 2022). A summary of the data is shown in Table 1.

To annotate the 1150 unique phosphoproteins that were identified in the phosphoproteome, we determined their potential functions based on ortholog mapping using the eggNOG-mapper (hit e-values ≤ 1 × 10^−3^) [40]. Of these, 1129 were recognized by the mapper as belonging to the Amoebozoa group and *E. histolytica* species, of which 813 fit into a protein cluster of orthologous groups (COG) with functional annotations (Supplemental Table S2, sheets 2 and 3). These annotations included regulation of RNA biosynthesis and transcription (COG categories A:57, J:57, and K:42); signal transduction mechanisms and trafficking (COG categories T: 144, U:50); posttranslational modifications, protein turnover, and chaperones (COG categories O:53); DNA replication and repair, chromatin structure, and chromosome partitioning (COG categories L:20, D:15, and B:22); transport and metabolism of carbohydrates, lipids, coenzymes, amino acids, and ions (COG categories G:23, P:17, E:15, I:14, F:10, H:3); cytoskeleton (COG categories Z and TZ:57); and other COG categories (C:9, V:4, W:3, M:2, Q:2). In addition, 194 proteins had a recognizable domain, but their function was unknown (COG category S) (Figure 6A). The functional analysis of molecular functions by GO term enrichment (813 proteins) indicates that the most enriched categories in the identified phosphoproteome were signalling, cytoskeleton functions, chromatin, and phosphatases (Supplemental Table S2, sheet 5 and Supplemental Figure S1). 

From the 723 more abundant peptides, 495 unique proteins were identified, including 323 with increased abundance and 172 with diminished abundance (FC > 2) (Supplemental Table S3, sheet 1). Of these, 344 had a COG category identifier (hit e-values ≤ 1 × 10^−3^) (Supplemental Table S3, sheets 2, 3 and 4), with representation in almost all COG categories of the phosphoproteome (Figure 6B). These proteins included those involved in the regulation of RNA biosynthesis and transcription (COG categories A:23, J:21, and K:13); signal transduction mechanisms and trafficking (COG categories T: 73, U:10); post-translational modifications, protein turnover, and chaperones (COG categories O:17); DNA replication, repair, chromatin structure, and chromosomes partition (COG categories L:7, D:3, and B:12); transport and metabolism of carbohydrates, lipids, coenzymes, amino acids, and ions (COG categories G:11, P:4, E:3, I:4, F:6, and H:1); cytoskeleton (COG categories Z and TZ:28), and other COG categories (C:5, V:3, W:1, M:0, and Q:0). In addition, 99 proteins had a recognizable domain but with unknown function (COG category S). These data are summarized in Table 1 and Figure 6C. The same categories as the global phosphoproteome were modulated upon Wtmn treatment (e.g., cytoskeleton, signal transduction mechanisms/trafficking, or DNA features) except for M and Q subsets, which were not found.

### 2.5. Phosphorylated Proteins Involved in Signalling Functions in E. histolytica 

To determine the potential functions of proteins within the phosphoproteome, we first focused our analysis on the components of signalling mechanisms, as they are tightly linked to the mechanobiome. For this analysis, we summarized data from the COG categories T, U and V, which have a link to signalling functions, in a single file (Supplemental Table S2, sheet 4). We also manually added proteins from other categories for which we identified at least one domain under the keywords kinase, phosphatase, or GTPase. The final file consisted of 290 phosphorylated proteins related to signalling functions in amoebae (Supplemental Table S4, sheet 1). The first analysed group consisted of 159 proteins whose phosphorylation was not modified by the Wtmn treatment (Supplemental Table S4, sheet 2). These proteins were submitted to tests for GO annotation and STRING functional enrichment analysis (FE) (Supplemental Table S4, sheets 3, 4, and 5). Among these protein sets, kinases were significantly enriched (37 proteins, FE = 3.54; *p* = 0.03). These included five proteins with transmembrane domains: one homologue to PI3K, two members of the TMK family (Tmk39 and TMK65) [41], and two unknown proteins, EHI_101280 and EHI_110250. The set of protein kinases was then sorted by STRING into three clusters which identified proteins containing diverse functional domains in addition to the kinase domain (CK, FHA, CRIB, WD40, CDK, MAP, LRR, Alpha-K, AN, LRR, KELCH, RIO, and ENDO-V) as well as proteins with unknown functions (Supplemental Table S4, sheet 4). Thymidine kinase and pantothenate kinase were also identified, although they were not present in the highly enriched GO set. 

The enrichment in GTPases was also significant in the GO terms analysis (17 proteins, FE: 2.63; *p* = 2.11 × 10^−4^) (Supplemental Table S4, sheet 6). These GTPases included dynamin and the G protein alpha subunit, as well as 15 small GTPase such as Rac2, Rho, two Ras, eleven Rabs, and nine activating protein GAPs specifics for Rap, Arf, Ras, Rab, or Rho. The third group with significant FE corresponded to phosphatases (FE: 2.15; *p*-value = 4.30 × 10^−4^), which included seven proteins containing the Calcineurin-like phosphoesterase domain, five from the protein phosphatase 2C family, two inositol polyphosphate 5-phosphatases, and myotubularin (Supplemental Table S4, sheet 7). Lastly, among the set of 159 proteins which were not modified by Wtmn treatment and did not cluster following the GO terms analyses, we identified components of endocytic functions, including 23 proteins involved in vesicular trafficking, essentially in endocytosis and Golgi-ER transition (Supplemental Table S4, sheet 8), including AP−1,2,3; Coatomers α, γ, δ; Snare, Snf, Sec1, and Sec7. 

The second group of analysed proteins carrying signalling functions corresponded to those with differential phosphorylation dynamics upon Wtmn treatment; this set consisted of 131 members (77 with increased (in) levels and 54 with diminished (dim) levels) (Supplemental Table S5, sheet 1). According to the InterPro and Pfam annotations of the domains, several protein groups were distinguished, including signal pathways regulated by small GTPases (38 in and 24 dim), kinases (21 in and 18 dim), phosphatases (10 in and 4 dim), and vesicles trafficking (3 in and 4 dim), which were the most significant candidates. Among the 14 phosphatases, we highlighted 3-phosphoinositide phosphatase (Pten) and inositol polyphosphate 5-phosphatase, which upregulated phosphorylation status in the presence of Wtmn. The 39 identified kinases were further analysed using STRING (Supplemental Table S5, sheet 2), and one kinase with increased phosphorylation was a homologue of the non-receptor protein-tyrosine kinase PTK2B, EHI_08344. This kinase regulates numerous signalling pathways, including activation of Rho GTPases, PI3K-AKT1, MAPK signalling cascades, and production of cGMP. Three TMKs were also identified (TMK46 was in, TMK40 and TMK54 were dim); in addition, PI4,5K and PI3,4K showed lower phosphorylation. All the protein kinases identified in this study were catalogued in interactive groups using STRING analysis (Supplemental Figure S2).

In summary, among the phosphorylated proteins with signalling functions, we identified 76 proteins with kinase activity, 88 with GTPase-related functions, 29 with phosphatases, and 28 linked to endocytic or ER-Golgi pathways. In addition, their phosphorylation sites were also identified. 

### 2.6. Phosphoproteome Proteins Involved in Cytoskeletal Functions

The second functional analysis of the phosphoproteome focused on cytoskeleton-linked proteins, which are important components of the mechanobiome. Cytoskeleton networks generate internal forces that allow cells to move and change shape and to connect cells (physically and chemically) to their external environment. In total, 53 proteins linked to the cytoskeleton were identified in the phosphoproteome (COG categories Z + TZ). Of these, 26 did not demonstrate phosphorylation changes upon Wtmn treatment, whereas 27 were modified significantly (Supplemental Table S6, sheet 1 and 2). Proteins involved in focal adhesion dynamics, actin binding, microfilament organization, and microtubule regulation were phosphorylated (Supplemental Table S6, sheet 1). Regarding FA elements and podosome structure, we found two proteins (EHI_080740 and EHI_167130) sharing significant homology with *Dictyostelium discoideum* filopodin/talin (approximately 22% identity). FERM domain, phosphotyrosine binding domain, and I/LWEQ domain were present in EHI_080740. In addition to these domains, two internal repeats were also shared with EHI_167130. The phosphorylation of these two proteins was shown to be upregulated by Wtmn (Supplemental Table S6, sheet 3).

Talin is enriched in focal adhesion sites of eukaryotic cells [42,43] and connects integrins to actin filaments following its interaction with vinculin and α-actinin, both of which were also identified in our analysis. For instance, vinculin EHI_083620 showed decreased phosphorylation, whereas in α-actinin, EHI_199000 phosphorylation was increased (Supplemental Table S6, sheet 2 and 3). Although the presence of integrins is not clearly demonstrated in *E. histolytica*, the data are of interest because vinculin links integrins to the cytoskeleton by alternatively binding talin or α-actinin and is involved in anchoring F-actin to the membrane. Actinins function as antiparallel dimers that simultaneously crosslink actin filaments and bind to integrins [44]. Talin and α-actinin have been shown to bind competitively to β3 integrin in fibronectin-adherent fibroblasts, with talin being involved in initial adhesion and α-actinin replacing talin to facilitate FAs maturation [45]. 

Another important member of the FA protein family is paxillin, which connects structural components to signalling components (e.g., tyrosine and serine/threonine kinases and GAPs/GEFs) [46]. We found that paxillin EHI_022960 (containing 4 LIM domains) is less phosphorylated in the presence of Wtmn. This is interesting because loss of paxillin phosphorylation has been proposed to impede FA disassembly to support FA maturation to fibrillar adhesions [47].

In addition to the components of adhesion structures described above, other members of the amoebic cytoskeleton were also identified in our proteomic analysis (Supplemental Table S6, sheet 1). Their phosphorylation abundance status was analysed in addition to the identification of their specific phosphorylation sites (Supplemental Table S6, sheets 2 and 3). Actin was found to interact with several classes of actin-binding proteins. A total of 17 candidates were identified (e.g., cortexillin, filamin, fimbrin, myosin II, villidin, gelsolin, and villin) and were involved in microfilament networking. In this category, the EHI_003930 protein carrying the I/LWEQ domain was found, which has homology with Huntingtin-interacting protein1-Sla2. It bundles actin filaments and mediates associations between actin- and clathrin-coated structures at the plasma membrane and trans-Golgi network. A total of 16 proteins were identified to be involved in microfilament dynamics (ARP2/3 members ARPC1 and ARPC3, orphans ARP, formin, profilin, cofilin, coactosin, actobinding, actophorin, and myosin IB). Seven proteins provided information on phosphorylation during microtubule dynamics, including β-tubulin, tubulin-associated factors, atg8, and kinesin.

The final group of cytoskeleton-related proteins identified in this proteomic analysis were LIM domain-containing proteins (Lin-11, Isl1, MEC-3) that are involved in multiple cellular mechanosensitive pathways [1]. LIM proteins are important to the mechanobiome because they sense stressed cytoskeletal networks by directly binding to a stretched conformation of the actin filament. This force-sensing mechanism by LIMs is conserved from yeast to mammals [48]. In addition to paxillin (containing 4 LIM domains), we recognized eight LIM proteins and their phosphorylation statuses. In the presence of Wtmn, two LIM candidates, LIM EHI_110280 and EHI_158150, showed increased and decreased phosphorylation, respectively. 

The identification of numerous phosphorylated cytoskeleton-related proteins in this amoebic proteome correlated well with the classes of proteins well-known in eukaryotes through their participation in cellular adhesive structures and cell motility.

### 2.7. Cellular Analysis of Proteins from Adhesive Structures in E. histolytica

Mechanical forces in cells are mediated by actin polymerization at the leading edge as well as by substrate-adherent structures. Understanding the interplay between F-actin levels and the activity of microfilament regulators can provide insights into the assembly and permanence of F-actin-enriched adhesive structures in *E. histolytica* [14].

Concerning actin, three phosphorylated peptides were identified in this work (Supplemental Table S6, sheet 3); two of them were more abundant in cells treated with Wtmn and were phosphorylated at Thr-204 and Ser-233. These two amino acids are located in subdomain IV of actin, which is the most flexible region of actin in *E. histolytica*, including residues 190–220 and 225–250 [14]. The third peptide had a reduced level of phosphorylation at Ser-366 and was in the S1 domain of actin. These phosphorylation sites are in addition to those previously described for actin in *E. histolytica* (e.g., Tyr-54, Thr-163, Tyr-167, Tyr-241, Ser-156 and Thr-107) [49,50].

Among the F-actin regulators, the ARP2/3 complex is a marker for podosomes, while paxillin clearly localizes to focal adhesion structures and has also been observed associated with podosomes. In *E. histolytica*, the ARP2/3 complex has been implicated in the formation of adhesion plates [14] and phagosomes [51]. In this work, ARPC1/p41 (phosphorylated at Thr-302) and ARPC3/p21 (4-fold increase in phosphorylated Ser-128) were identified in addition to three orphan ARPs (ARPv2, ARPv1, and ARPi). ARPC1 and APRC3 are involved in cell adhesion and motility [52,53], and in *E. histolytica*, ARPC1 is recruited to the phagosome initiation site following its phosphorylation by the kinase EhAK1 [54]. However, the potential role of these diverse ARPs in amoebic motility has not yet been described. The functions of paxillin in *E. histolytica* are not known, but in the phosphoproteome, we found a 4-fold reduction in two phosphorylated peptides at Thr-212 and Ser-58. 

**Figure 7:**
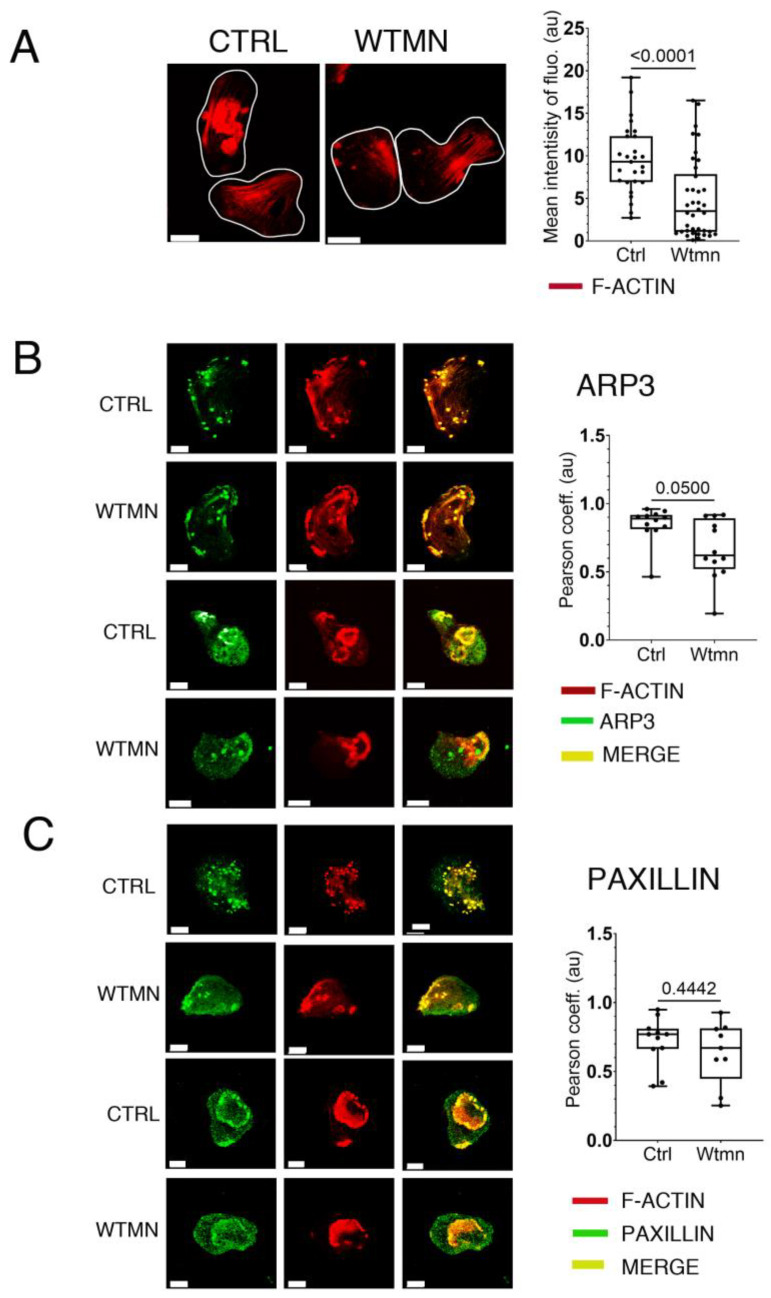
F-actin content and F-actin colocalization with markers of adhesive structure. (**A**) Intensity of F-actin. Fluorescence measurements were obtained from confocal micrographs of trophozoites in which F-actin was decorated with TRITC-phalloidin (red). The entire amoeba was considered as an independent ROI, and cell segmentation was performed using the active contours plugin in Icy, allowing the automatic assessment of F-actin mean intensity of fluorescence for each cell. Wtmn treatment significantly decreased the mean intensity of F-actin. Scale bar = 10 µm. (**B**) Localization of ARP3 and F-actin. Two sample cells are shown. Adhesive structures for control and Wtmn-treated cells were immuno-labelled for the detection of ARP3 (green) and detection of F-actin staining with TRITC-phalloidin (red). Independent adhesive structures were selected as ROIs, and levels of colocalization between ARP3 and F-actin were quantified with the “colocalization Studio” plugin of the Icy software. Wtmn-treated trophozoites (*n* = 12, 0.62 au) were compared to controls (*n* = 12, 0.89 au). Wtmn treatment significantly decreased the level of colocalization between ARP3 and F-actin (*p* = 0.05) by 30%. Scale bar = 10 µm, au = arbitrary units. (**C**) Localization of paxillin and F-actin. Two sample cells are shown. Adhesive structures for control or Wtmn-treated cells were immunolabelled for the detection of paxillin (green) and decoration of F-actin with TRITC-phalloidin (red). Independent adhesive structures were selected as ROIs, and levels of colocalization between Paxillin and F-actin were quantified using the “Colocalization Studio” plugin of the Icy software. Wtmn treated trophozoites (*n* = 9, 0.67 au) were compared to the controls (*n* = 11, 0.77 au). Wtmn treatment decreased level of colocalization between paxillin and F-actin by 13% (*p* = 0.4). Scale bar = 10 µm, au = arbitrary units.

Altogether, these proteomic findings encouraged us to use confocal microscopy to examine the F-actin content of Wtmn-treated trophozoites and the association of microfilaments with the ARP2/3 complex or paxillin in subcellular structures. For this purpose (Figure 7), we used antibodies specific to *E. histolytica* to detect ARP3, a central subunit of the ARP2/3 complex [14], and prepared an anti-paxillin antibody recognizing C4LUK9 (EHI_022960) described in the methods section and Supplemental Figure S3. F-actin content was determined using quantitative fluorescence imaging (Figure 7A), and the data showed that the level of F-actin was reduced by 62.4% (*p* = 0.0001) in Wtmn-treated trophozoites (*n* = 38, 3.5 au) compared to control trophozoites (*n* = 27, 9.3 au). This finding was supported by changes observed in the adhesive structures of trophozoites that retained dots and plates in the presence of Wtmn, as evidenced by alterations in their ARP3 and paxillin content. For ARP3, F-actin colocalization was significantly reduced by 30% (*p* = 0.05), and paxillin was reduced by 13% (*p* = 0.44) (Figure 7C). The reduced F-actin levels and altered F-actin ratios with ARP3 or paxillin indicate that the phosphorylation changes observed in the proteome upon disruption of PI3K activity by Wtmn lead to changes in microfilament dynamics that could impact the renewal of adhesive structures in *E. histolytica*.

## 3. Discussion

A detailed knowledge of signalling processes, including kinases, is essential for understanding cell motility, particularly in the context of responses due to environmental changes during the invasion of human tissues by the pathogenic parasite *E. histolytica*. Previous data suggest that PI3Ks are relevant to amoebic motility, although the proteins targeted by these kinases and their phosphorylation sites are largely unknown. In mammalian cells, PI3K isoforms fall into three classes based on structural features and lipid substrate preferences. However, in *E. histolytica*, PI3K class II is absent [55]. Nevertheless, Wtmn, which is a potent inhibitor of amoebic PI3Ks, has a clear effect on signalling pathways activated during amoebic displacements [25,34,56].

To study changes in cell morphology and motility, and at the same time identify components of PI3K-influenced signalling pathways, we used a sublethal dose of 3 nM of Wtmn. This treatment induced significant changes in amoeba morphology, reflected in a reduction of cell polarization, although trophozoites remained adherent to the substrate. Therefore, this phenotype indicates an important impact of PI3Ks on protein phosphorylation and microfilament dynamics. Indeed, inhibition of PI3Ks correlated with the reduction of F-actin levels, which led to a decrease in the ratio of F-actin to ARP3 or paxillin. In turn, this decreased the number of adhesive structures.

Given the complex composition and function of podosomes [6], many other components of the cytoskeleton are expected to participate in maintaining amoebae polarization, since ARP3 and paxillin are structural elements of the core and ring elements of podosomes [57]. Among these cytoskeleton-related phosphorylated proteins, an important data set of candidates for amoebic adhesive structure dynamics was identified here. Cataloguing the dataset with bioinformatics tools allowed for better annotation of 53 cytoskeleton proteins that are known to be members of diverse functional cellular compartments, including adhesive structures (5), microfilament networking (17), microfilament turnover (16), microtubule dynamics (7), and potential regulators of actin-rich cytoskeletal dynamics such as the LIM proteins (8).

All these factors are of interest when considering the cytoskeletal dynamics of adhesive structure architectures during environmental changes. For example, when cells grow on stretched supports (such as glass), F-actin mostly organizes into bundles by linking actin filaments together. This work established the features of actin-bundling proteins, and their phosphorylation state, such as α-actinin, fimbrin, and filamin. PI3K-dependent phosphorylation of these proteins in *E. histolytica* is relevant because (i) the cap of podosomes is composed of cross-linked and bundled actin microfilaments, and (ii) stress fibbers, which consist of bundles of 10 to 30 actin filaments, are present in the cell division furrow along with myosin II [58], and at the back of polarized amoebae [14], where filamin 2 (ABP-120) [23] and α–actinin concentrates [20]. 

Actin microfilaments in amoebae are sensitive to mechanical forces [34], and similar to other cells, it is expected that *E. histolytica* adapts the configuration of microfilaments to balance external and internal forces [59]. LIM-domain-containing proteins (e.g., zyxin and paxillin) are involved in stabilizing stressed actin filaments [48] and in cellular functions related to generating and responding to mechanical forces [1,60]. In this study, eight proteins containing LIM domains (2 to 5 LIMs) were identified, three of which (EHI_001070, EHI_096420, and EHI_161940) were previously found to be associated with F-actin [14]. The presence of LIM proteins, together with actin-bundling proteins, paxillin, and ABP, provides opportunities for mechanosensing studies in *E. histolytica*. 

The presence of Wtmn also impacts the kinetic parameters of moving amoebae by altering the phosphorylation changes and slowing down the cell velocity. Amoeboid cells extend pseudopods to guide their displacements, which are not random. Depending on cytoskeletal actomyosin activity, the extension of pseudopodia can be promoted in the cell front, giving cells a tendency to move persistently in the same direction, referred to as “memory” behaviour [61]. The data indicate that the superdiffusive mode of *E. histolytica* motion is preserved upon treatment with a low dose of Wtmn [39]. However, in previous work, we observed that the application of external forces at the rear pole of moving *E. histolytica* induced cell polarization with a frontal pseudopod, which strongly increased directional motion [34]. However, this phenomenon was abolished by the treatment of amoebae with high doses of Wtmn (100 nM), which converted motility from directional to random [34]. We can conclude that PI3Ks activates a finely tuned biological signalling response based on the level of external stimuli. The transduction and amplification of specific external stimuli require the discovery of surface receptors for these mechanical signals. 

In addition to PI3Ks, *E. histolytica* has a large number of protein kinases that make up approximately 3% of the total proteome [62]. We identified 76 proteins carrying a kinase domain and their phosphorylated peptides, including five members of the amoebic transmembrane kinase family (TMK39, TMK40, TMK46, TMK54, and TMK65). Among these proteins, two had phosphorylation statuses that were altered in the presence of Wtmn (TMK46 was increased and TMK54 was reduced). The TMKs of *E. histolytica* share similarities with serine/threonine and tyrosine kinases, and may play a role in the recognition and signalling of various stimuli [63]. One potential receptor candidate is TMK-54, which influences both the growth of amoebae and the localization of the heavy chain of the lectin Gal-GalNAc on the surface of trophozoites [41]. This discovery is interesting because the Gal-GalNAc lectin concentrates in sites where amoebae adhere to mammalian cells [64]. The carboxyl end of the lectin heavy chain interacts with the cytoskeleton [65] via a bundling spectrin-like protein [66], and amoebic strains affected in lectin activities are unable to move in three-dimensional environments (e.g., liver tissue), whereas their displacements are normal on two-dimensional surfaces (e.g., glass or surface intestinal cells in culture) [39]. A possible synergy between the signalling functions of TMK54 and Gal/GalNAc lectin in the control of amoebic motility is possible, although it remains to be studied. Further signalling pathways necessary for PI3K-derived signalling include the small GTPases and phosphatases, both of which were abundantly identified in the proteomics analysis.

## 4. Materials and Methods

### 4.1. Entamoeba histolytica Culture and Live Cells Imaging

Trophozoites of the *Entamoeba histolytica* strain HM-1: IMSS were cultivated at 37 °C in a glass tube filled with TYI-S-33 medium [67]. The medium was discarded and the trophozoites were washed with TYI-S-33 medium devoid of serum and vitamins (incomplete medium) previously warmed to 37 °C. The cells were gently tapped to resuspend them and were counted under a microscope. Next, 10^4^ amoebae were seeded on 35 mm glass-bottomed imaging dishes (Ibidi, Nanterre, France) and filled with incomplete TYI-S-33 medium. The dishes were incubated for 2 h at 37 °C in anaerobic conditions using a GENbag anaerobic device (Ref. 45534 Biomerieux, Marcy-l’Étoile, France). Wortmannin (Ref. W1628, Sigma, Taufkirchen, Germany) was added at a final concentration of 3 nM to one set of amoebae and incubated for 30 min. After incubation, migrating cells were recorded using a spinning disk microscope UltraVIEW VoX (Perkin Elmer, Waltham, MA, USA), with an objective of 10× and temperature control set to 37 °C. Images were acquired (1 image·s^−1^ or 1 image.1.5 s^−1^ according to experiment) using Volocity three-dimensional image analysis software (Perkin Elmer, USA).

### 4.2. Image Analysis to Determine the Kinetic Parameters of E. histolytica

All image analyses were conducted using the open-source Icy software v2.4.2 (http://icy.bioimageanalysis.org, accessed on 11 November 2021) [37]. Bright field sequences of migrating cells were obtained as described above using a spinning disk confocal microscope. Tracking of migrating cells was performed manually using the Manual Tracking plugin in Icy. The quantification of the average speed was automatically performed by the Motion Profiler in the Track Manager processor. Sequences of 65 frames starting from the 85th time point of the video microscopy were selected using the Time Clip processor and submitted to the Motion Profiler, selecting the options “use real units” and “show speed instead of displacement”. The resulting data were exported in an excel file (Microsoft^R^) for further plotting and statistical analysis using Prism GraphPad version 9.5.1, Boston, MA, USA.

To determine the mean square displacements (MSD), image sequences obtained for 65 s (65 or 43 frames, depending on the recording speed) were submitted to the Track processor MSD in Icy, which includes improved statistical classification for tracking dynamics [68]. The obtained values of both MSD and the time points for each track were exported in excel files from the Output console of the Icy interface. Each movie (14 movies from control cells, *n* = 227 and 13 movies from Wtmn treated cells, *n* = 166) was treated independently to obtain the slope of tracks (α) curves as follows. First, a table was built in excel with the exported MSD values and the corresponding time points. A first MSD threshold was established at 800 µm^2^. Second, the plot derived from the table (Prism GraphPad Software version 9.5.1, USA, Boston, MA, USA) was converted to X and Y coordinates in a logarithmic scale with an MSD threshold of 2.5. Third, after determining the simple linear regression for each MSD plot, the slope value α was provided. The α factor reflects the occurring motion dynamics. Since α = 1 corresponds to Brownian motion, α > 1 reflects the superdiffusion motion, and α < 1 reflects the subdiffusive motion [68,69]. Alpha values derived from each track curve (MSD vs. t) were plotted, and the median α value with statistical *p*-value of confidence was obtained using Prism GraphPad Software version 9.5.1, Boston, MA, USA.

### 4.3. Image Analysis to Determine Trophozoites Morphology

Brightfield images of fixed amoebae were obtained using a confocal microscope (LSM700, Zeiss, Jena, Germany), with a 63× objective and NA 1.4. To measure the length, width, and roundness of the amoebae, a region of interest (ROI) was drawn manually around each cell corresponding to an ellipse, and the precise detection of the borders was automatically determined using the Active Contour plugin in Icy. The first and second axis of the ellipse were used to determine the major (length) and minor (width) axis of the cell, and the roundness was also selected as a criterion. The resulting data were exported as an excel file for each ROI, and the results were plotted and statistically analyzed using Prism GraphPad Software version 9.5.1, Boston, MA, USA. 

### 4.4. Enumeration of Cell Adhesion Structures

Actin-rich structures were annotated in fixed trophozoites labelled with TRITC-phalloidin. Confocal images were acquired using a Zeiss LSM 700 laser scanning microscope (Objective 10×, NA 0.5). Confocal planes were acquired in the region nearest to the bottom of the cells, 1 µm away from the glass. Actin-rich structures were quantified in trophozoites from 30 confocal planes, which were individually annotated and manually sorted within each ROI according to the following nomenclature: empty cell (Line), cell containing a cluster of dots (Point), and cell containing an adhesive plate (Polygon). A cell containing several similar structures was annotated once, but a cell containing several different structures was annotated for each structure. After annotation, the Icy software summarized the data from the ROIs. The cell sorting results were exported into an Excel file for data analysis. Each ROI group of amoebae was converted to a percentage corresponding to one image. The percentages of amoebae in each image were plotted using Prism GraphPad Software version 9.5.1, Boston, MA, USA. 

### 4.5. Immunofluorescence Staining of ARP3 and Paxillin

Trophozoites (4.5 × 10^4^) were incubated anaerobically on slides for 12 h at 37 °C in TYI-S-33 medium using a GENbag anaerobic device (Ref. 45534, Biomerieux, Marcy-l’Étoile, France). The medium was removed, and the cells were fixed at room temperature for 30 min with PBS buffer containing 4% PFA (Ref. P6148, Sigma-Aldrich Merck, Darmstadt, Germany). The fixed trophozoites were permeabilized with 0.05% Triton X-100 in PBS for 3 min. The slides were then washed with PBS and quenched with 50 mM NH_4_Cl (Ref. A9434, Sigma-Aldrich Merck, Germany) for 15 min. The samples were blocked with 2% BSA (A2153, Sigma-Aldrich Merck, Germany) for 1 h. The slides were incubated with the primary antibody for 1 h at room temperature, washed with PBS twice, and incubated with the secondary antibody for 30 min at room temperature.

The primary antibodies used in this work included a rabbit polyclonal anti-ARP3 antibody generated in our laboratory (1:200 dilution) [14] and a polyclonal anti-paxillin antibody (1:200 dilution) raised in rabbits (Eurogentec, Seraing, Belgium) by immunisation with the purified peptides 162-KPKPTPQPAQKKDDL and 325-PFPTPSFFQKDGNPY from the amino acid sequence of *E. histolytica* paxillin protein (locus tag: EHI_022960 and UniProt ID: C4LUK9). The specificity of the anti-paxillin antibody (1:500 dilution) was confirmed using western blotting according to [14] and showed a unique protein of 51 kDa (Supplementary Figure S3). The secondary antibody was goat anti-rabbit Alexa Fluor-488 (Molecular Probes, Eugene, OR, USA, 1:200 dilution). To decorate microfilaments, we used tetramethylrhodamine B isothiocyanate (TRITC) crosslinked phalloidin at a 1:200 dilution (Ref. P1951, Sigma Aldrich Merck, Darmstadt, Germany). Lastly, the slides were washed with BSA-free PBS and mounted with ProLong antifading reagent containing 4′,6-diamidino-2-phenylindole DAPI (Molecular Probes, USA). Confocal images were acquired using a Zeiss LSM 700 laser scanning microscope.

### 4.6. F-actin Levels and Colocalization with ARP3 or Paxillin

The intensity level of F-actin in trophozoites was determined for micrographs of F-actin (labelled with TRITC-phalloidin) obtained using a Zeiss LSM700 confocal microscope (Objective 63×, NA 1.4). These images were taken from three-dimensional stacks of confocal planes (15 planes, 1 µm slices) from the bottom to the central region of the cell body. An ROI covering the entire cell was drawn manually, and the precise detection of the borders was automatically determined using the Active Contour plugin in Icy. The mean intensity of fluorescence was exported as an excel file for each ROI. The results were plotted, and statistical tests were performed using Prism GraphPad Software, version 9.5.1, Boston, MA, USA. 

Colocalization of F-actin with the actin partner was determined using the “Colocalization Studio” plugin in Icy software, providing Pearson’s correlation coefficients with *p*-values < 0.05. The strengths of associations were considered weak (PS = 0.1–0.3), medium (PS = 0.3–0.5), or strong (PS = 0.5–1.0). Specific ROIs within the cells (i.e., adhesion plates and clusters of dots) were obtained from raw image data of 44 randomly selected trophozoites. 

### 4.7. Statistical Tests for Image Analysis

All measurements for control or Wtmn-treated cells were compared using Prism GraphPad Software using a non-parametric unpaired Mann-Whitney statistical test. 

### 4.8. Wortmannin Treatment and Preparation for Proteomics of Whole Cell Extracts

Log phase growing trophozoites from *Entamoeba histolytica* strain HM-1: IMSS were incubated with 3 nM of wortmannin (Ref. W1628, Sigma, Germany) for 30 min, and whole cell extracts were prepared from the untreated and treated amoebae (3 biological replicates for each condition) as follows: the trophozoites from each experimental sample (7 × 10^6^ cells) were washed with cold (4 °C) TBS (0.2 M Tris, 1.5 M NaCl), harvested by shaking, centrifuged, and resuspended in Guanidine Lysis Buffer (6 M Gn-HCl plus 0.1 M Tris pH 8.0). Then, they were extensively sonicated on ice for 10 min with the intervals required to avoid overheating the samples. The resulting lysates were centrifuged at 15,000 rpm for 20 min to remove insoluble debris, and clarified supernatant protein concentration was estimated using the BCA method. 

### 4.9. Samples of Proteins Preparation and Phosphopeptide Enrichment 

For sample preparation, 5 mg of Gn-HCl protein lysates were first reduced using 5 mM TCEP (tris(2-carboxyethyl) phosphine), then further alkylated with 50 mM iodoacetamide. The alkylated proteins were further diluted using 50 mM ammonium bicarbonate to achieve a final Gn-HCl concentration of 0.6 M, then they were digested with trypsin (1:50, trypsin: lysate ratio, Promega) for 16 h at 37 °C. The overnight digests were clarified with a brief spin, and the clean supernatant pH was adjusted to approximately pH 2 using 10% Trifluoroacetic Acid (TFA). Sep-Pak (Waters, Milford, MA, USA) columns were washed with methanol and washing buffer (2% Acetonitrile/0.1% TFA), followed by loading of total peptides. The column was washed 3 times with washing buffer (2% Acetonitrile/0.1% TFA), and the final peptide elution was performed using high acetonitrile-containing elution buffers (50% and 80% Acetonitrile/0.1%TFA). The peptide mixture was dried using a speed vacuum. The dried peptide pellet was dissolved in Phthalic acid buffer (0.1% Phthalic Acid/20% Water/80% Acetonitrile, 2.5% TFA) and mixed with TiO_2_ (Titansphere 5 μm, GL sciences, Torrance, CA, USA) beads on a rotator for two h. The beads were washed twice with Phthalic acid buffer, followed by washing with 80% acetonitrile/0.1% TFA, and finally 0.1% TFA. Phosphopeptides were eluted using 0.3 M NH_4_OH, and the pH was adjusted approximately 2 using 50% TFA. The Phosphopeptides were dried using a speed vacuum and further clarified using C18 columns (ThermoFisher Scientific, Waltham, MA, USA) as per the above-mentioned buffers.

### 4.10. Mass-Spectrometry (MS) Analysis and Data Acquisition

The purified and dried phosphopeptide pellet was resuspended in Buffer-A (5% Acetonitrile/0.1% Formic acid). For the mass spectrometry analysis of the peptide mixtures, all the experiments were performed using an EASY-nLC1000 system (ThermoFisher Scientific, USA) coupled to a QExactive mass spectrometer (ThermoFisher Scientific, USA) equipped with a nanoelectrospray ion source. A total of 1 μg of the phosphopeptide mixture was loaded and resolved using a self-packed column (2 µm resin, 75 µm ID, 50 cm length, prepared using a laser fiber puller, Sutter Instruments, Novato, CA, USA). A column oven (Sonation GmBH, Biberach, Germany) was used to maintain a constant temperature of 60 °C to reduce backpressure during the nanoLC run. The peptides were loaded into a nanocolumn with Buffer A (5% Acetonitrile/0.1% Formic acid), then eluted with the following Buffer B (95% Acetonitrile/0.1% Formic acid) gradients: 2–5% (5 min), 5–10% (40 min), 10–30% (110 min), 30–60% (20 min), and 80% (5 min) at a flow rate of 250 nL/min for a total run time of 180 min. The QExactive was operated using the Top10 HCD data-dependent acquisition mode with a full scan resolution of 70,000 at *m/z* 400, AGC target 3e6, a max injection time (IT) of 20 ms, and a scan range of 300–1700 *m/z* in profile mode. MS/MS scans were acquired at a resolution of 17,500 at *m/z* 400, AGC target 1e6, a max injection time (IT) of 60 ms, scan range 200–2000 *m/z*, and an isolation window of 1.6 *m/z* in profile mode with an HCD fragmentation NCE value of 27 and a dynamic exclusion of 30 s. The lock mass option was enabled for polydimethylcyclosiloxane (PCM) ions (*m/z* = 445.120025) for internal recalibration during the run. 

### 4.11. Mass-Spectrometry Data Processing and Statistical Analysis 

All the six raw files (3 sets of each Wtmn-unstimulated and stimulated samples) were analysed using MaxQuant software (v1.6.3.4) (www.maxquant.org/maxquant/, accessed on 20 April 2021) against the *E. histolytica* UniProt reference proteome (7959 proteins) database (www.uniprot.org, accessed on 3 May 2021). For the Andromeda search, the precursor and fragment mass tolerances were set at 10 ppm and 20 ppm, respectively. The specificity of the protease used to generate peptides was set for Trypsin/P along with a maximum missed cleavages value of two. Carbamidomethyl (C) on cysteine was used as a fixed modification, and Phosphorylation (P) on Serine, Threonine, Tyrosine, oxidation (O) of methionine, and N-terminal acetylation were considered as variable modifications for the database search. The settings also included reverse sequences of the database and 247 built-in contaminants in the default settings of MaxQuant. For peptide identification, a peptide posterior error probability (PEP) threshold of 0.05 was specified. Both PSM and protein FDR were set to 0.01. Default settings were applied for all other parameters. For relative quantitative comparison among the biological replicates of the two conditions, label-free quantification with “match between runs” option was utilized within the MaxQuant suite, with standard settings for generating the peak lists from 6 raw files (Xcalibur, ThermoFisher Scientific, USA) and a retention time alignment window of 1 min. The protein groups were created using the default settings of MaxQuant in case the peptide sets were common among multiple proteins. 

The data matrix containing phosphopeptides (3411) was imported in Perseus software (v1.6.2.3), and the phosphopeptides were filtered for reverse and contaminant entries. For further analysis, a high quality phospho site localization probability of 0.75 was considered. All the filtered, high-quality respective phosphopeptides were further analysed based on their relative label-free quantification (LFQ) values. The resulting phosphopeptides were further filtered for entries where LFQ values were available in at least 2 bioreplicates among the two study groups (total of 3 bioreplicates for both wortmannin-unstimulated and stimulated samples). The resulting 2352 phosphorylated peptides were considered to be part of the *E. histolytica* phosphoproteome. For quantitative studies, the LFQ abundance values of these filtered peptides (2352) were log2 transformed, and imputation was applied using perseus default settings (width 0.3, downshift 1.8), where missing values were replaced by random numbers that were drawn from a normal distribution. A student’s *t* test was applied to the Wtmn-stimulated and unstimulated group samples using a *p*-value significance threshold level of 0.05, and the test results were represented as volcano plot. Z-score normalization was applied using median abundance values, and student’s *t*-test significant phospho-peptide (*n* = 723) abundance values were used for hierarchical clustering of rows and/or columns (distance: Euclidean, linkage: average, preprocess: k-means, number of clusters: 300, maximum iterations: 10) in order to generate a heatmap/Clustergram. The mass spectrometry proteomics data have been deposited into the ProteomeXchange Consortium (https://www.proteomexchange.org/, accessed on 14 April 2023) via the PRIDE partner repository with the dataset identifier PXD040787 with free access.

### 4.12. EggNOG Data Mining and Detailed Category Annotation

The set of phosphorylated peptides (2352) corresponded to 1150 unique proteins with an accession ID in UniProt database (https://www.uniprot.org/, accessed on 21 November 2021). The unique protein sets were further submitted to search for orthologous groups in the EggNOG database V.5 [70] using the eggNOG-mapper V.2 (http://eggnog-mapper.embl.de/, accessed on 1 April 2022) according to their defined parameters (hit e-values ≤ 1 × 10^−3^). One-letter abbreviations for functional category correspondence can be found at https://www.ncbi.nlm.nih.gov/research/cog#, accessed on 2 April 2022. When needed, datasets were combined using the Venn diagram approach (https://bioinfogp.cnb.csic.es/tools/venny/, accessed on 22 April 2021). For further functional annotation, the predicted proteins of Entamoeba were related to the protein classes, and gene ontology enrichments were revealed through a search performed with the following Amoeba DB tools: search for gene ID and data analysis for GO terms (molecular functions) (https://amoebadb.org/amoeba/app, accessed on 22 April 2021). In some cases, STRING (https://string-db.org/, accessed on 26 April 2021) [71] clustering methods were used. For protein homologies search we used CLUSTAL Omega (1.2.4) multiple sequence alignment tool [72].

## 5. Conclusions

Our work evaluated the global status of the phosphoproteome, as well as the modifications of protein phosphorylation after inhibiting the activity of PI3Ks in the in vitro cultured *E. histolytica*. For the first time, 1150 unique proteins carrying at least one phosphorylated peptide were identified in this study. Among these, upon PI3Ks inhibition, our results show changes in the phosphorylation of cytoskeleton-related proteins, leading to a significant reduction in the number of amoebic adhesive structures, altering cell morphology and velocity. Several candidate proteins are thought to have a function when the parasite recognizes changes in the chemical and topographic composition of the external environment, such as the different compartments of the intestinal tissue. In addition to protein phosphorylation of mechanobiome components, other phosphorylated proteins involved in the biology and lifestyle of *E. histolytica* have been highlighted. Taken together, our work indicates that changes in phosphorylation have potential clinical applications in blocking *E. histolytica*, as the parasite’s efficient tissue invasion depends on its motility and cytoskeleton-driven cytopathic effects.

## Figures and Tables

**Table 1:** Summary of diverse categories obtained using eggNOG mapping. See Figure 6 legend for explanations.

All Phosphoproteome (Control)		
Description of Global Biological Categories	COGs (Number)	Total
Signal transduction mechanisms and trafficking	T: 144, U:50	194
Unknown function	S:194	194
RNA biosynthesis and transcription	A:57, J:57, K:42	156
Transport and metabolism of carbohydrates, lipids, coenzymes, amino acids, and ions	G:23, P:17, E:15, I:14, F:10, H:3	82
DNA replication, repair, chromatin structure, and chromosomes partition	L:20, D:15 and B:22	57
Cytoskeleton	Z and TZ:57	57
Posttranslational modifications, protein turnover, and chaperones	O:53	53
Other	C:9, V:4, W:3, M:2, Q:2	20
TOTAL		813
Wortmannin treatment		
Description of global biological categories	COG (number)	Total
Signal transduction mechanisms and trafficking	T: 73, U:10	83
Unknown function	S:99	99
RNA biosynthesis and transcription	A:23, J:21, K:13	57
Transport and metabolism of carbohydrates, lipids, coenzymes, amino acids, and ions	G:11, P:4, E:3, I:4, F:6, H:1	29
DNA replication, repair, chromatin structure, and chromosomes partition	L:7, D:3 and B:12	22
Cytoskeleton	Z and TZ:28	28
Posttranslational modifications, protein turnover, and chaperones	O:17	17
Other	C:5, V:3, W:1 M:0, Q:0	9
TOTAL		344
Ratio Wortmannin/Control		
Description of global biological categories		Percentage
Signal transduction mechanisms and trafficking		46
Unknown function		51
RNA biosynthesis and transcription		37
Transport and metabolism of carbohydrates, lipids, coenzymes, amino acids, and ions		35
DNA replication, repair, chromatin structure, and chromosomes partition		39
Cytoskeleton		49
Posttranslational modifications, protein turnover, and chaperones		32
Other		45

## Data Availability

The datasets generated in the present study are available through the ProteomeXchange Consortium (https://www.proteomexchange.org/, accessed on 14 April 2023) via the PRIDE partner repository with the dataset identifier PXD040787 with free access.

## Supplementary Materials

The following supporting information can be downloaded at: https://www.mdpi.com/article/10.3390/ijms24108726/s1.
