# Supplementary material for: Live Cells Imaging and Comparative Phosphoproteomics Uncover Proteins from the Mechanobiome in Entamoeba histolytica
Source: Int J Mol Sci. 2023 May 13;24(10):8726. doi: 10.3390/ijms24108726 (PMC10217974; doi:10.3390/ijms24108726)
Supplement: Supplementary file 1 [file ijms-24-08726-s001.zip › Supplemental material.docx]

Live cells imaging and comparative phosphoproteomics uncover proteins from the mechanobiome in *Entamoeba histolytica*

Gagan D. Jhingan ^1, #^, Maria Manich ^1,2^, Jean-Christophe Olivo-Marin ^3,4^ and Nancy Guillen ^1,2, 4 *^

^1^ Institut Pasteur, Cell Biology of Parasitism Unit, 75015 Paris, France.

^2^ Institut Pasteur, Biological Image Analysis Unit, 75015 Paris, France.

^3^ Centre National de la Recherche Scientifique, CNRS UMR3691, 75015 Paris, France.

^4^ Centre National de la Recherche Scientifique, CNRS-ERL9195, 75015 Paris, France.

SUPPLEMENTAL MATERIAL

**FIGURES**


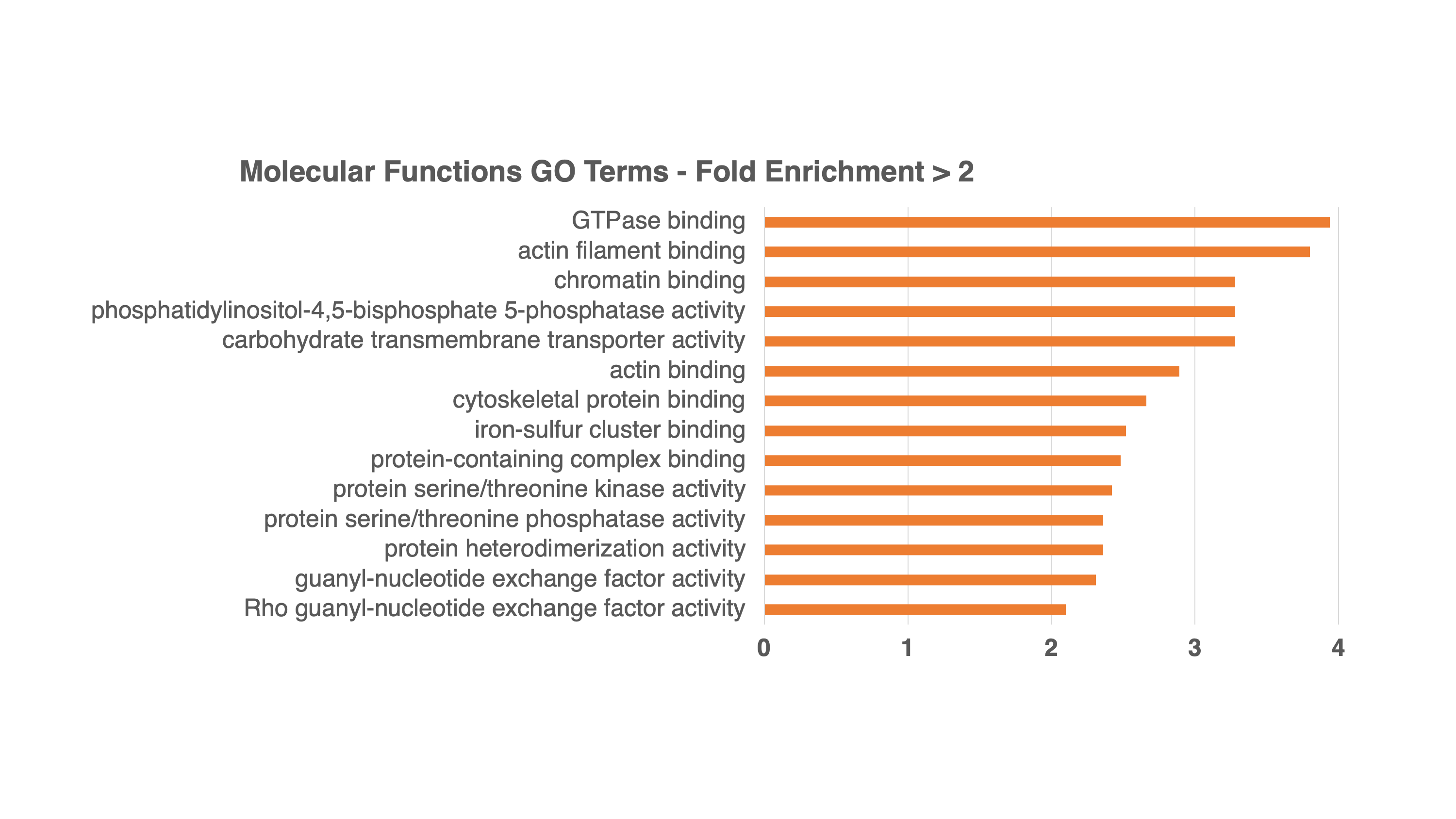


**Supplemental Figure 1. GO term enrichment in the phosphoproteome from *E. histolytica***.

AmoebaDB tools (https://amoebadb.org) were used to test identified phosphoproteins data for GO term enrichment of the gene set relative to the background list of all the genes in the amoeba genome. The graph shows the fold enrichment (FE) for molecular function GO Terms with an FE ≥ 2.5. The summary of gene ontology analysis is provided in Table 2 sheet 5.


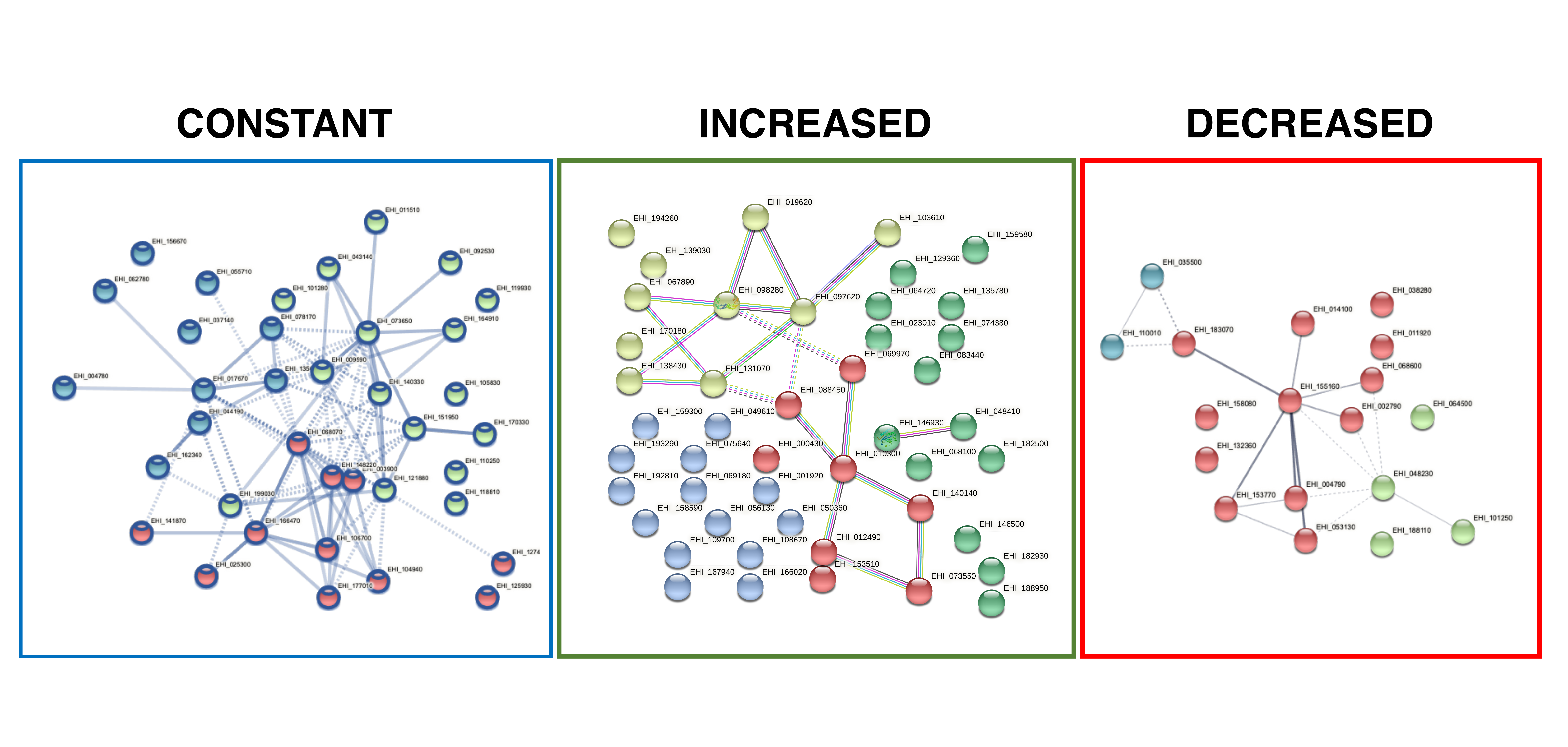


**Supplemental Figure 2. Interactive protein–protein association network for the identified kinases.** The list of proteins presenting a kinase domain identified in this study was catalogued in an interactive protein–protein association network using STRING software (https://string-db.org/) for gene functions clustering (K-means clustering methods). Proteins with constant abundance from Table 5, sheet 4 and 5 (left panel, 3 clusters) refers to proteins whose values does not change in the presence of Wtmn. Proteins which abundance change in the presence of Wtmn (Table 6 sheet 2) refer to two classes: these with phosphorylation augmentation in the presence of Wtmn (middle panel, 4 clusters), and these with decrease abundance in Wtmn treatment (right panel, 3 clusters).


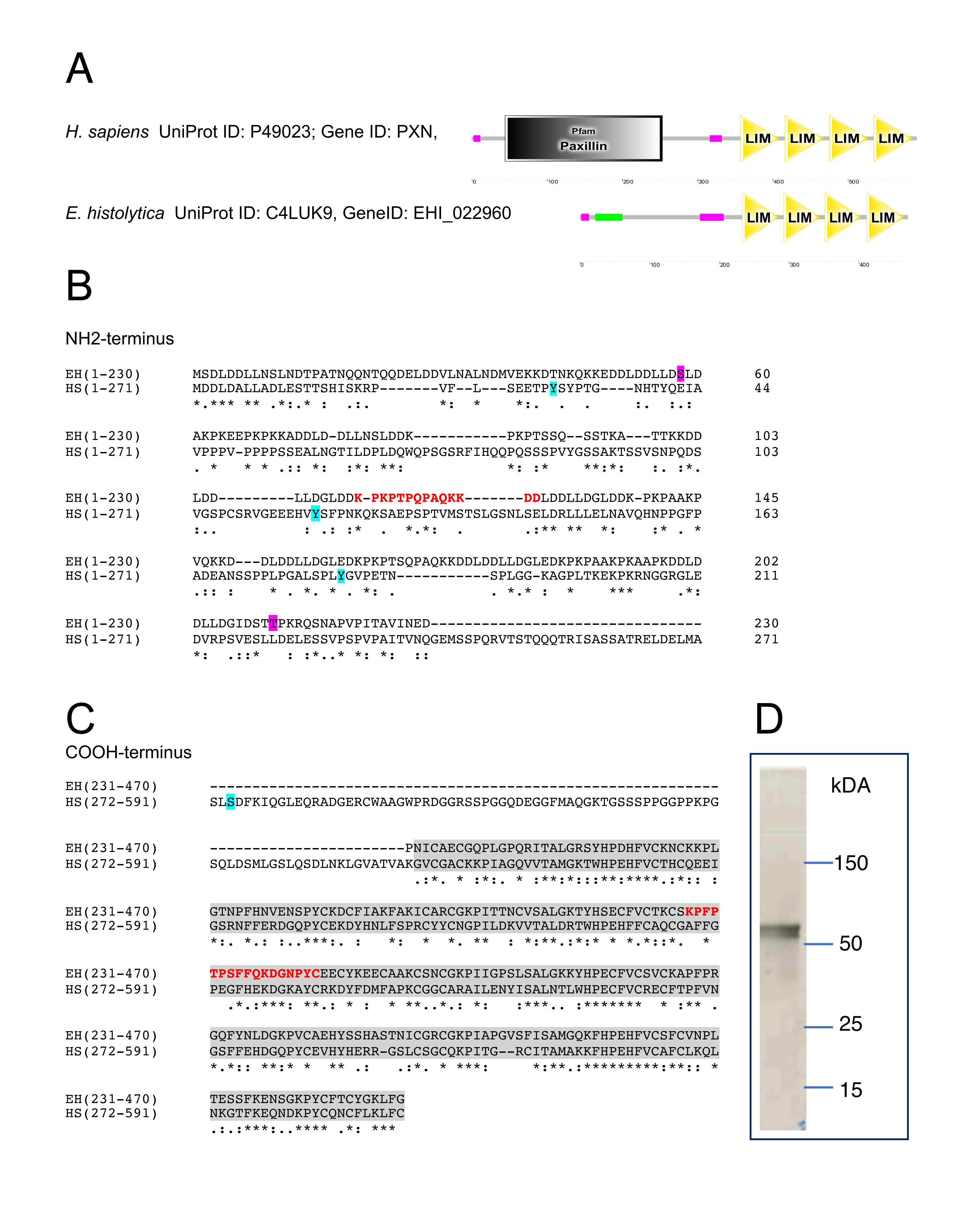


**Supplemental Figure 3. Paxillin in *E. histolytica*.**

A- The diagram was obtained by comparing the amino acid sequences in SMART (http://smart.embl-heidelberg.de/), it represents the paxillin domains in *H. sap*iens (P49023) and *E. histolytica* (C4LUK9). The yellow triangles correspond to the LIM domains.

B- Proteins homology at the NH2-terminal domains obtained with CLUSTAL Omega (1.2.4) multiple sequence alignment [42]. The identity found was 25.37%

C- Proteins homology at the COOH-terminal domains obtained with CLUSTAL Omega (1.2.4) multiple sequence alignment [42]. The identity found was 41.35%.

The grey region defined the LIM domains. Paxillin phosphorylated peptides were underlined in *E. histolytica* (magenta) and in *H. sapiens* (green)

D- Western blot of a crude extract of trophozoites from *E. histolytica* (10e5 cells) incubated with an anti-paxillin antibody. Peptides highlighted in red in the amino acid sequence of *E. histolytica* were used for the immunization of rabbits (Eurogentec, Belgium). A unique peptide of 55 kDa in molecular mass has been identified.

TABLES

[Table S1: All proteomics data.xlsx](Supplemental%20Tables/Table%201.%20All%20proteomics%20data.xlsx)

[Table S2: Phosphoproteome-EHI.xlsx](Supplemental%20Tables/Table%202.Phosphoproteome-EHI.xlsx)

[Table S3: Fold changes and Volcano Plot.xlsx](Supplemental%20Tables/Table%203.%20FC-VolcanoPlot.xlsx)

[Table S4: Proteins from the phosphoproteome with signalling activities.xlsx](Supplemental%20Tables/Table%204.%20Signalling.xlsx)

[Table S5: Signalling proteins with modified phosphorylation abundance.xlsx](Supplemental%20Tables/Table%205.%20Signalling%20WtmnFC2.xlsx)

[Table S6: Proteins from the cytoskeleton found phosphorylated.xlsx](Supplemental%20Tables/Table%206.%20CYTOSKELETON.xlsx)

VIDEOS

[Video 1. Supplemental Movie1-Control cells.avi](Supplemental%20Movie1-Control%20cells.avi)

Migrating cells incubated at 37°C were recorded with a spinning disk microscope objective 10x. Images were acquired (1 image.sec-1) and treated with Volocity 3D image analysis software. The resulting video was made with 120 microscopy frames, time interval for reproduction of the film is of 100 msec per frame.

[Video 2. Supplemental Movie2-Wortmannin cells.avi](Supplemental%20Movie%202-Wortmannin%20cells.avi)

Migrating cells incubated at 37°C were recorded with a spinning disk microscope objective 10x. Images were acquired (1.5 image.sec-1) and treated with Volocity 3D image analysis software. The resulting video was made with 120 microscopy frames, time interval for reproduction of the film is of 100 msec per frame.
